# 1-Tosyl-2-[(1-tosyl-1*H*-benzimidazol-2-yl)methyl­sulfanyl]-1*H*-benzimidazole

**DOI:** 10.1107/S1600536811011822

**Published:** 2011-04-07

**Authors:** Nassir N. Al-Mohammed, Yatimah Alias, Zanariah Abdullah, Hamid Khaledi

**Affiliations:** a Department of Chemistry, University of Malaya, 50603 Kuala Lumpur, Malaysia

## Abstract

In the title compound, C_29_H_24_N_4_O_4_S_3_, the two *N*-tosyl­benzimidazolyl unit are connected through a —S—CH_2_— fragment, the dihedral angle between the benzimidazole rings being 76.09 (5)°. The methyl­thio group is disordered with respect to exchange of the S and C atoms in a 0.547 (4):0.453 (4) ratio. In the crystal, C—H⋯O and C—H⋯π inter­actions connect adjacent mol­ecules into infinite layers parallel to the *ab* plane. The crystal packing is further stabilized by a π–π inter­action [centroid–centroid separation = 3.5187 (4) Å].

## Related literature

For the structures of similar compounds, see: Hayashi *et al.* (2008 ▶); Rashid *et al.* (2006 ▶, 2007 ▶).
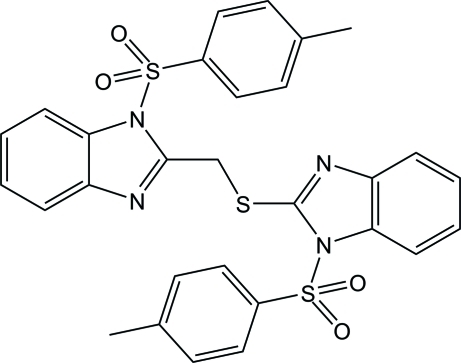


## Experimental

### 

#### Crystal data

C_29_H_24_N_4_O_4_S_3_
*M* *_r_* = 588.70
Triclinic, 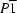

*a* = 8.2524 (6) Å
*b* = 13.5905 (10) Å
*c* = 13.8117 (10) Å
α = 62.5191 (8)°
β = 75.4090 (9)°
γ = 85.9930 (9)°
*V* = 1327.99 (17) Å^3^
*Z* = 2
Mo *K*α radiation
μ = 0.32 mm^−1^
*T* = 100 K
0.38 × 0.35 × 0.21 mm

#### Data collection

Bruker APEXII CCD diffractometer
Absorption correction: multi-scan (*SADABS*; Sheldrick, 1996 ▶) *T*_min_ = 0.887, *T*_max_ = 0.935
8528 measured reflections
4762 independent reflections
4405 reflections with *I* > 2σ(*I*)
*R*_int_ = 0.017

#### Refinement

*R*[*F* ^2^ > 2σ(*F* ^2^)] = 0.039
*wR*(*F* ^2^) = 0.117
*S* = 1.05
4762 reflections
382 parameters
4 restraints
H-atom parameters constrained
Δρ_max_ = 0.29 e Å^−3^
Δρ_min_ = −0.41 e Å^−3^

### 

Data collection: *APEX2* (Bruker, 2007 ▶); cell refinement: *SAINT* (Bruker, 2007 ▶); data reduction: *SAINT*; program(s) used to solve structure: *SHELXS97* (Sheldrick, 2008 ▶); program(s) used to refine structure: *SHELXL97* (Sheldrick, 2008 ▶); molecular graphics: *X-SEED* (Barbour, 2001 ▶); software used to prepare material for publication: *SHELXL97* and *publCIF* (Westrip, 2010 ▶).

## Supplementary Material

Crystal structure: contains datablocks I, global. DOI: 10.1107/S1600536811011822/pv2404sup1.cif

Structure factors: contains datablocks I. DOI: 10.1107/S1600536811011822/pv2404Isup2.hkl

Additional supplementary materials:  crystallographic information; 3D view; checkCIF report

## Figures and Tables

**Table 1:** Hydrogen-bond geometry (Å, °) *Cg*1 and *Cg*2 are the centroids of the C8–C13 and C23–C28 rings, respectively.

*D*—H⋯*A*	*D*—H	H⋯*A*	*D*⋯*A*	*D*—H⋯*A*
C6—H6⋯O4^i^	0.95	2.48	3.314 (3)	147
C20—H20⋯O3^ii^	0.95	2.46	3.386 (3)	164
C25—H25⋯*Cg*1^iii^	0.95	2.99	3.744 (3)	138
C15—H15*A*⋯*Cg*2^iv^	0.99	2.98	3.62 (2)	124

Symmetry codes: (i) 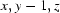; (ii) 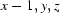; (iii) 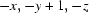; (iv) 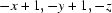.

**Fig. 1.**
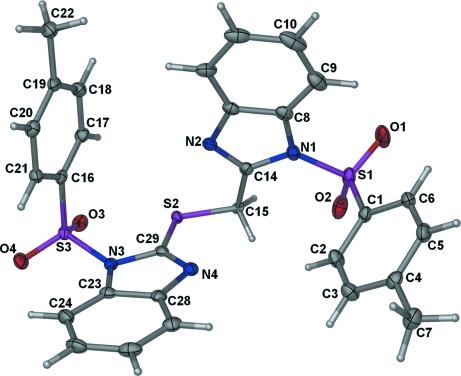
The molecular structure of the title compound showing displacement ellipsoids at the 50% probability level. Hydrogen atoms are drawn as spheres of arbitrary radius. Only the major component of the disordered methylthio group is depicted.

## supplementary crystallographic information

### Comment

The title compound (Fig. 1) is the *N*-tosylation product of 2-(thiomethyl-2'-benzimidazolyl)-benzimidazole. The two benzimidazolyl rings are connected through the methythio fragment, making a dihedral angle of 76.09 (5)°. The two *N*-bound tosyl groups and the benzimidazolyl rings subtend angles of 103.77 (10)° at S1 and 104.48 (10)° at S3 atoms. These values are comparable to those reported for similar structures (Hayashi *et al.*, 2008; Rashid *et al.*, 2006; Rashid *et al.*, 2007). In the crystal, intermolecular C—H···O and C—H···*π* interactions link the adjacent molecules into polymeric layers parallel to the *ab* plane. The crystal packing is further stabilized by a *π—π* interaction formed by the six-membered rings (C8—C13) of anti-parallelly arranged benzimidazole rings related by symmetry of -*x*, -*y*, -*z* + 1 [centroids separation = 3.5187 (4) Å]. Moreover, intramolecular C—H···O and C—H···N hydrogen bonding occurs (Table 1).

### Experimental

Sodium (0.17 g) was added to a solution of 2-mercaptobenzimidazole (1 g, 6.7 mmol) in anhydrous methanol (20 ml) and the mixture was stirred at room temperature for 20 minutes. To the mixture, 2-chloromethylbenzimidazole (1.11 g, 6.67 mmol) was added dropwise under vigorous stirring, and then left to stir overnight. The solvent was removed under reduced pressure and the remaining liquid was washed with water and crystallized from tetrahydrofuran (THF) to give the white solid of 2-(thiomethyl-2'-benzimidazolyl)-benzimidazole. A solution of *p*-toluene sulfonyl chloride (0.75 g, 3.91 mmol) in pyridine (5 ml) was added dropwise to a solution of 2-(thiomethyl-2'-benzimidazolyl)-benzimidazole (0.5 g, 1.78 mmol) in pyridine (5 ml) at 273 K, within 2 hr. The mixture was stirred at room temperature overnight and then poured into a beaker containg 100 ml ice water. It was then stirred for another 15 minutes, extracted with dichloromethane and washed with distilled water (3 x 10 ml). The organic layer was dried with magnesium sulfate and evaporated. The obtained solid was recrystallized from toluene to give the colorless crystals of the title compound.

### Refinement

Hydrogen atoms were placed at calculated positions at distances C—H = 0.95, 0.98 and 0.99 Å for aryl, methyl and methylene type H-atoms, respectively, and were treated as riding on their parent atoms, with U*iso*(H) = 1.2–1.5 times U*eq*(C). S2—C15 fragment was found to be disordered over two positions. From anisotropic refinement, the major component of the disorder had a site occupancy factor of 0.547 (4). The corrsponding bond distances involving the disordred groups were restrained to be equal by the SADI command in *SHELXL97* (Sheldrick, 2008).

### Crystal data

**Table:**

C_29_H_24_N_4_O_4_S_3_	*Z* = 2
*M**_r_* = 588.70	*F*(000) = 612
Triclinic, *P*1	*D*_x_ = 1.472 Mg m^−^^3^
Hall symbol: -P 1	Mo *K*α radiation, λ = 0.71073 Å
*a* = 8.2524 (6) Å	Cell parameters from 6545 reflections
*b* = 13.5905 (10) Å	θ = 2.6–29.6°
*c* = 13.8117 (10) Å	µ = 0.32 mm^−^^1^
α = 62.5191 (8)°	*T* = 100 K
β = 75.4090 (9)°	Block, colorless
γ = 85.9930 (9)°	0.38 × 0.35 × 0.21 mm
*V* = 1327.99 (17) Å^3^	

### Data collection

**Table:**

Bruker APEXII CCD diffractometer	4762 independent reflections
Radiation source: fine-focus sealed tube	4405 reflections with *I* > 2σ(*I*)
graphite	*R*_int_ = 0.017
φ and ω scans	θ_max_ = 25.3°, θ_min_ = 2.6°
Absorption correction: multi-scan (*SADABS*; Sheldrick, 1996)	*h* = −9→8
*T*_min_ = 0.887, *T*_max_ = 0.935	*k* = −15→16
8528 measured reflections	*l* = −16→16

### Refinement

**Table:**

Refinement on *F*^2^	Primary atom site location: structure-invariant direct methods
Least-squares matrix: full	Secondary atom site location: difference Fourier map
*R*[*F*^2^ > 2σ(*F*^2^)] = 0.039	Hydrogen site location: inferred from neighbouring sites
*wR*(*F*^2^) = 0.117	H-atom parameters constrained
*S* = 1.05	*w* = 1/[σ^2^(*F*_o_^2^) + (0.0548*P*)^2^ + 1.7481*P*] where *P* = (*F*_o_^2^ + 2*F*_c_^2^)/3
4762 reflections	(Δ/σ)_max_ < 0.001
382 parameters	Δρ_max_ = 0.29 e Å^−^^3^
4 restraints	Δρ_min_ = −0.41 e Å^−^^3^

### Special details

**Table:**

Geometry. All e.s.d.'s (except the e.s.d. in the dihedral angle between two l.s. planes) are estimated using the full covariance matrix. The cell e.s.d.'s are taken into account individually in the estimation of e.s.d.'s in distances, angles and torsion angles; correlations between e.s.d.'s in cell parameters are only used when they are defined by crystal symmetry. An approximate (isotropic) treatment of cell e.s.d.'s is used for estimating e.s.d.'s involving l.s. planes.
Refinement. Refinement of *F*^2^ against ALL reflections. The weighted *R*-factor *wR* and goodness of fit *S* are based on *F*^2^, conventional *R*-factors *R* are based on *F*, with *F* set to zero for negative *F*^2^. The threshold expression of *F*^2^ > σ(*F*^2^) is used only for calculating *R*-factors(gt) *etc*. and is not relevant to the choice of reflections for refinement. *R*-factors based on *F*^2^ are statistically about twice as large as those based on *F*, and *R*- factors based on ALL data will be even larger.

### Fractional atomic coordinates and isotropic or equivalent isotropic displacement parameters (Å^2^)

**Table:**

	*x*	*y*	*z*	*U*_iso_*/*U*_eq_	Occ. (<1)
S1	0.38609 (8)	0.01814 (5)	0.23234 (5)	0.02441 (16)	
S2	0.4294 (5)	0.3694 (3)	0.2743 (3)	0.0161 (5)	0.547 (4)
C15	0.445 (2)	0.2467 (15)	0.257 (2)	0.020 (4)	0.547 (4)
H15A	0.4941	0.2670	0.1768	0.024*	0.547 (4)
H15B	0.5227	0.1974	0.3007	0.024*	0.547 (4)
S2'	0.4759 (8)	0.2429 (4)	0.2489 (6)	0.0158 (7)	0.453 (4)
C15'	0.419 (3)	0.3702 (15)	0.2508 (16)	0.032 (4)	0.453 (4)
H15C	0.3390	0.3536	0.3238	0.039*	0.453 (4)
H15D	0.5210	0.4076	0.2474	0.039*	0.453 (4)
S3	0.22947 (7)	0.59437 (4)	0.24425 (5)	0.01762 (15)	
O1	0.3429 (3)	−0.09759 (15)	0.30145 (16)	0.0368 (5)	
O2	0.5483 (2)	0.06264 (16)	0.21509 (16)	0.0325 (4)	
O3	0.3524 (2)	0.55663 (14)	0.30806 (14)	0.0240 (4)	
O4	0.2006 (2)	0.70985 (13)	0.18860 (15)	0.0247 (4)	
N1	0.2505 (2)	0.08379 (15)	0.29227 (16)	0.0194 (4)	
N2	0.1401 (2)	0.21679 (15)	0.33908 (15)	0.0176 (4)	
N3	0.2886 (2)	0.55596 (15)	0.14188 (15)	0.0171 (4)	
N4	0.3248 (2)	0.42440 (15)	0.08180 (16)	0.0184 (4)	
C1	0.3376 (3)	0.05944 (19)	0.10314 (19)	0.0196 (5)	
C2	0.3707 (3)	0.1693 (2)	0.0204 (2)	0.0245 (5)	
H2	0.4191	0.2222	0.0334	0.029*	
C3	0.3317 (3)	0.2000 (2)	−0.0811 (2)	0.0258 (5)	
H3	0.3539	0.2748	−0.1382	0.031*	
C4	0.2606 (3)	0.1234 (2)	−0.1015 (2)	0.0217 (5)	
C5	0.2307 (3)	0.0143 (2)	−0.0176 (2)	0.0248 (5)	
H5	0.1836	−0.0389	−0.0307	0.030*	
C6	0.2683 (3)	−0.01874 (19)	0.0852 (2)	0.0238 (5)	
H6	0.2470	−0.0937	0.1421	0.029*	
C7	0.2154 (3)	0.1571 (2)	−0.2115 (2)	0.0275 (5)	
H7A	0.2293	0.0947	−0.2295	0.041*	
H7B	0.2890	0.2206	−0.2717	0.041*	
H7C	0.0985	0.1779	−0.2049	0.041*	
C8	0.0772 (3)	0.05618 (19)	0.33644 (18)	0.0209 (5)	
C9	−0.0229 (3)	−0.0310 (2)	0.3535 (2)	0.0291 (6)	
H9	0.0219	−0.0873	0.3336	0.035*	
C10	−0.1929 (4)	−0.0311 (2)	0.4016 (2)	0.0347 (7)	
H10	−0.2666	−0.0886	0.4139	0.042*	
C11	−0.2576 (3)	0.0506 (2)	0.4321 (2)	0.0301 (6)	
H11	−0.3740	0.0469	0.4659	0.036*	
C12	−0.1562 (3)	0.1371 (2)	0.41446 (19)	0.0245 (5)	
H12	−0.2009	0.1931	0.4350	0.029*	
C13	0.0131 (3)	0.13913 (18)	0.36571 (18)	0.0186 (5)	
C14	0.2774 (3)	0.18328 (18)	0.29586 (18)	0.0173 (4)	
C16	0.0379 (3)	0.51977 (18)	0.32649 (18)	0.0158 (4)	
C17	0.0298 (3)	0.42479 (19)	0.42717 (19)	0.0205 (5)	
H17	0.1282	0.3987	0.4532	0.025*	
C18	−0.1247 (3)	0.36822 (19)	0.48967 (19)	0.0215 (5)	
H18	−0.1316	0.3031	0.5591	0.026*	
C19	−0.2696 (3)	0.40555 (18)	0.45200 (19)	0.0191 (5)	
C20	−0.2574 (3)	0.50082 (19)	0.34960 (19)	0.0198 (5)	
H20	−0.3552	0.5264	0.3227	0.024*	
C21	−0.1050 (3)	0.55858 (18)	0.28654 (19)	0.0185 (5)	
H21	−0.0977	0.6237	0.2171	0.022*	
C22	−0.4367 (3)	0.3441 (2)	0.5213 (2)	0.0242 (5)	
H22A	−0.5272	0.3933	0.4979	0.036*	
H22B	−0.4454	0.3201	0.6012	0.036*	
H22C	−0.4463	0.2789	0.5099	0.036*	
C23	0.2228 (3)	0.59687 (18)	0.04581 (18)	0.0180 (5)	
C24	0.1482 (3)	0.69526 (19)	−0.0111 (2)	0.0235 (5)	
H24	0.1322	0.7512	0.0130	0.028*	
C25	0.0988 (3)	0.7073 (2)	−0.1043 (2)	0.0270 (5)	
H25	0.0469	0.7731	−0.1450	0.032*	
C26	0.1228 (3)	0.6259 (2)	−0.1402 (2)	0.0260 (5)	
H26	0.0863	0.6368	−0.2043	0.031*	
C27	0.1992 (3)	0.5289 (2)	−0.08383 (19)	0.0227 (5)	
H27	0.2167	0.4736	−0.1088	0.027*	
C28	0.2491 (3)	0.51520 (18)	0.01016 (18)	0.0178 (5)	
C29	0.3434 (3)	0.44894 (18)	0.15870 (18)	0.0169 (4)	

### Atomic displacement parameters (Å^2^)

**Table:**

	*U*^11^	*U*^22^	*U*^33^	*U*^12^	*U*^13^	*U*^23^
S1	0.0326 (3)	0.0210 (3)	0.0251 (3)	0.0125 (2)	−0.0141 (3)	−0.0131 (3)
S2	0.0151 (7)	0.0142 (7)	0.0176 (12)	0.0017 (5)	−0.0032 (8)	−0.0068 (6)
C15	0.015 (7)	0.018 (4)	0.016 (5)	0.010 (3)	−0.002 (4)	−0.002 (3)
S2'	0.0114 (15)	0.0158 (12)	0.0196 (14)	0.0021 (10)	−0.0037 (12)	−0.0078 (9)
C15'	0.036 (8)	0.032 (5)	0.026 (8)	−0.008 (4)	0.007 (4)	−0.017 (5)
S3	0.0140 (3)	0.0182 (3)	0.0225 (3)	0.0000 (2)	−0.0041 (2)	−0.0110 (2)
O1	0.0639 (14)	0.0200 (9)	0.0298 (10)	0.0163 (9)	−0.0213 (9)	−0.0112 (8)
O2	0.0270 (9)	0.0440 (11)	0.0452 (11)	0.0198 (8)	−0.0213 (8)	−0.0320 (10)
O3	0.0165 (8)	0.0313 (9)	0.0294 (9)	0.0016 (7)	−0.0076 (7)	−0.0174 (8)
O4	0.0230 (9)	0.0179 (8)	0.0325 (9)	−0.0025 (7)	−0.0036 (7)	−0.0122 (7)
N1	0.0219 (10)	0.0167 (9)	0.0209 (10)	0.0049 (7)	−0.0082 (8)	−0.0088 (8)
N2	0.0157 (9)	0.0158 (9)	0.0177 (9)	0.0000 (7)	−0.0035 (7)	−0.0050 (8)
N3	0.0153 (9)	0.0166 (9)	0.0171 (9)	0.0006 (7)	−0.0021 (7)	−0.0067 (8)
N4	0.0142 (9)	0.0180 (9)	0.0198 (9)	−0.0022 (7)	−0.0011 (7)	−0.0071 (8)
C1	0.0218 (12)	0.0194 (11)	0.0198 (11)	0.0059 (9)	−0.0062 (9)	−0.0110 (9)
C2	0.0289 (13)	0.0197 (12)	0.0288 (13)	−0.0003 (10)	−0.0117 (10)	−0.0120 (10)
C3	0.0284 (13)	0.0200 (12)	0.0263 (12)	0.0011 (10)	−0.0088 (10)	−0.0075 (10)
C4	0.0181 (11)	0.0273 (12)	0.0221 (12)	0.0065 (9)	−0.0041 (9)	−0.0146 (10)
C5	0.0266 (12)	0.0239 (12)	0.0290 (13)	0.0002 (10)	−0.0054 (10)	−0.0171 (11)
C6	0.0288 (13)	0.0168 (11)	0.0244 (12)	0.0006 (9)	−0.0027 (10)	−0.0102 (10)
C7	0.0286 (13)	0.0326 (14)	0.0274 (13)	0.0086 (11)	−0.0106 (11)	−0.0178 (11)
C8	0.0248 (12)	0.0201 (11)	0.0142 (10)	−0.0011 (9)	−0.0076 (9)	−0.0033 (9)
C9	0.0387 (15)	0.0221 (12)	0.0271 (13)	−0.0040 (11)	−0.0153 (11)	−0.0076 (10)
C10	0.0416 (16)	0.0285 (14)	0.0280 (13)	−0.0144 (12)	−0.0202 (12)	−0.0001 (11)
C11	0.0243 (13)	0.0377 (15)	0.0181 (12)	−0.0101 (11)	−0.0070 (10)	−0.0020 (11)
C12	0.0208 (12)	0.0291 (13)	0.0166 (11)	−0.0051 (10)	−0.0034 (9)	−0.0045 (10)
C13	0.0195 (11)	0.0170 (11)	0.0145 (10)	−0.0019 (9)	−0.0067 (9)	−0.0018 (9)
C14	0.0193 (11)	0.0161 (11)	0.0140 (10)	0.0029 (8)	−0.0052 (8)	−0.0045 (9)
C16	0.0141 (10)	0.0180 (11)	0.0194 (11)	0.0018 (8)	−0.0038 (8)	−0.0123 (9)
C17	0.0164 (11)	0.0234 (12)	0.0215 (11)	0.0046 (9)	−0.0048 (9)	−0.0105 (10)
C18	0.0224 (12)	0.0190 (11)	0.0191 (11)	0.0028 (9)	−0.0054 (9)	−0.0056 (9)
C19	0.0185 (11)	0.0176 (11)	0.0223 (11)	−0.0008 (9)	−0.0016 (9)	−0.0118 (9)
C20	0.0171 (11)	0.0233 (12)	0.0225 (11)	0.0039 (9)	−0.0071 (9)	−0.0127 (10)
C21	0.0176 (11)	0.0165 (11)	0.0203 (11)	0.0015 (9)	−0.0036 (9)	−0.0081 (9)
C22	0.0188 (12)	0.0233 (12)	0.0271 (12)	0.0012 (9)	−0.0030 (10)	−0.0102 (10)
C23	0.0138 (10)	0.0181 (11)	0.0148 (10)	−0.0026 (8)	−0.0014 (8)	−0.0023 (9)
C24	0.0208 (12)	0.0194 (12)	0.0234 (12)	0.0031 (9)	−0.0035 (9)	−0.0055 (10)
C25	0.0226 (12)	0.0248 (13)	0.0199 (12)	0.0019 (10)	−0.0055 (10)	0.0010 (10)
C26	0.0171 (11)	0.0336 (14)	0.0176 (11)	−0.0034 (10)	−0.0034 (9)	−0.0034 (10)
C27	0.0157 (11)	0.0273 (13)	0.0207 (11)	−0.0042 (9)	−0.0001 (9)	−0.0088 (10)
C28	0.0122 (10)	0.0180 (11)	0.0171 (10)	−0.0035 (8)	0.0004 (8)	−0.0044 (9)
C29	0.0115 (10)	0.0157 (11)	0.0163 (10)	−0.0014 (8)	0.0015 (8)	−0.0037 (9)

### Geometric parameters (Å, °)

**Table:**

S1—O1	1.4260 (19)	C7—H7A	0.9800
S1—O2	1.426 (2)	C7—H7B	0.9800
S1—N1	1.6761 (19)	C7—H7C	0.9800
S1—C1	1.750 (2)	C8—C9	1.386 (3)
S2—C29	1.758 (3)	C8—C13	1.394 (3)
S2—C15	1.781 (14)	C9—C10	1.393 (4)
C15—C14	1.521 (15)	C9—H9	0.9500
C15—H15A	0.9900	C10—C11	1.390 (4)
C15—H15B	0.9900	C10—H10	0.9500
S2'—C14	1.710 (6)	C11—C12	1.383 (4)
S2'—C15'	1.770 (14)	C11—H11	0.9500
C15'—C29	1.487 (14)	C12—C13	1.386 (3)
C15'—H15C	0.9900	C12—H12	0.9500
C15'—H15D	0.9900	C16—C17	1.383 (3)
S3—O3	1.4246 (17)	C16—C21	1.395 (3)
S3—O4	1.4274 (17)	C17—C18	1.390 (3)
S3—N3	1.6756 (19)	C17—H17	0.9500
S3—C16	1.753 (2)	C18—C19	1.394 (3)
N1—C8	1.408 (3)	C18—H18	0.9500
N1—C14	1.410 (3)	C19—C20	1.394 (3)
N2—C14	1.298 (3)	C19—C22	1.508 (3)
N2—C13	1.396 (3)	C20—C21	1.383 (3)
N3—C23	1.416 (3)	C20—H20	0.9500
N3—C29	1.422 (3)	C21—H21	0.9500
N4—C29	1.296 (3)	C22—H22A	0.9800
N4—C28	1.403 (3)	C22—H22B	0.9800
C1—C6	1.385 (3)	C22—H22C	0.9800
C1—C2	1.391 (3)	C23—C28	1.392 (3)
C2—C3	1.383 (3)	C23—C24	1.394 (3)
C2—H2	0.9500	C24—C25	1.383 (4)
C3—C4	1.395 (3)	C24—H24	0.9500
C3—H3	0.9500	C25—C26	1.392 (4)
C4—C5	1.387 (3)	C25—H25	0.9500
C4—C7	1.510 (3)	C26—C27	1.387 (3)
C5—C6	1.387 (3)	C26—H26	0.9500
C5—H5	0.9500	C27—C28	1.388 (3)
C6—H6	0.9500	C27—H27	0.9500
			
O1—S1—O2	120.60 (12)	C11—C10—H10	119.1
O1—S1—N1	106.15 (11)	C9—C10—H10	119.1
O2—S1—N1	105.55 (10)	C12—C11—C10	121.4 (2)
O1—S1—C1	108.87 (11)	C12—C11—H11	119.3
O2—S1—C1	110.46 (11)	C10—C11—H11	119.3
N1—S1—C1	103.77 (10)	C11—C12—C13	117.5 (2)
C29—S2—C15	96.0 (8)	C11—C12—H12	121.2
C14—C15—S2	113.2 (10)	C13—C12—H12	121.2
C14—C15—H15A	108.9	C12—C13—C8	120.6 (2)
S2—C15—H15A	108.9	C12—C13—N2	128.5 (2)
C14—C15—H15B	108.9	C8—C13—N2	110.8 (2)
S2—C15—H15B	108.9	N2—C14—N1	112.32 (19)
H15A—C15—H15B	107.8	N2—C14—C15	121.5 (6)
C14—S2'—C15'	97.1 (9)	N1—C14—C15	126.1 (6)
C29—C15'—S2'	115.7 (9)	N2—C14—S2'	128.0 (2)
C29—C15'—H15C	108.3	N1—C14—S2'	119.7 (2)
S2'—C15'—H15C	108.3	C15—C14—S2'	6.5 (7)
C29—C15'—H15D	108.3	C17—C16—C21	121.3 (2)
S2'—C15'—H15D	108.3	C17—C16—S3	120.80 (17)
H15C—C15'—H15D	107.4	C21—C16—S3	117.91 (17)
O3—S3—O4	120.56 (10)	C16—C17—C18	118.9 (2)
O3—S3—N3	106.26 (10)	C16—C17—H17	120.6
O4—S3—N3	105.40 (10)	C18—C17—H17	120.6
O3—S3—C16	109.64 (10)	C17—C18—C19	121.0 (2)
O4—S3—C16	109.22 (10)	C17—C18—H18	119.5
N3—S3—C16	104.48 (10)	C19—C18—H18	119.5
C8—N1—C14	106.02 (18)	C20—C19—C18	118.9 (2)
C8—N1—S1	124.86 (16)	C20—C19—C22	120.7 (2)
C14—N1—S1	128.62 (16)	C18—C19—C22	120.5 (2)
C14—N2—C13	106.02 (19)	C21—C20—C19	121.0 (2)
C23—N3—C29	105.04 (18)	C21—C20—H20	119.5
C23—N3—S3	124.58 (15)	C19—C20—H20	119.5
C29—N3—S3	124.38 (15)	C20—C21—C16	119.0 (2)
C29—N4—C28	105.61 (19)	C20—C21—H21	120.5
C6—C1—C2	121.3 (2)	C16—C21—H21	120.5
C6—C1—S1	119.01 (18)	C19—C22—H22A	109.5
C2—C1—S1	119.63 (18)	C19—C22—H22B	109.5
C3—C2—C1	118.6 (2)	H22A—C22—H22B	109.5
C3—C2—H2	120.7	C19—C22—H22C	109.5
C1—C2—H2	120.7	H22A—C22—H22C	109.5
C2—C3—C4	121.4 (2)	H22B—C22—H22C	109.5
C2—C3—H3	119.3	C28—C23—C24	122.1 (2)
C4—C3—H3	119.3	C28—C23—N3	105.19 (19)
C5—C4—C3	118.5 (2)	C24—C23—N3	132.7 (2)
C5—C4—C7	120.1 (2)	C25—C24—C23	116.6 (2)
C3—C4—C7	121.5 (2)	C25—C24—H24	121.7
C4—C5—C6	121.4 (2)	C23—C24—H24	121.7
C4—C5—H5	119.3	C24—C25—C26	121.9 (2)
C6—C5—H5	119.3	C24—C25—H25	119.0
C1—C6—C5	118.8 (2)	C26—C25—H25	119.0
C1—C6—H6	120.6	C27—C26—C25	121.0 (2)
C5—C6—H6	120.6	C27—C26—H26	119.5
C4—C7—H7A	109.5	C25—C26—H26	119.5
C4—C7—H7B	109.5	C26—C27—C28	117.9 (2)
H7A—C7—H7B	109.5	C26—C27—H27	121.0
C4—C7—H7C	109.5	C28—C27—H27	121.0
H7A—C7—H7C	109.5	C27—C28—C23	120.5 (2)
H7B—C7—H7C	109.5	C27—C28—N4	128.5 (2)
C9—C8—C13	122.5 (2)	C23—C28—N4	111.05 (19)
C9—C8—N1	132.7 (2)	N4—C29—N3	113.03 (19)
C13—C8—N1	104.79 (19)	N4—C29—C15'	120.7 (6)
C8—C9—C10	116.1 (3)	N3—C29—C15'	126.3 (6)
C8—C9—H9	121.9	N4—C29—S2	128.5 (2)
C10—C9—H9	121.9	N3—C29—S2	118.49 (19)
C11—C10—C9	121.8 (2)	C15'—C29—S2	7.8 (7)

### Hydrogen-bond geometry (Å, °)

**Table:**

Cg1 and Cg2 are the centroids of the C8–C13 and C23–C28 rings, respectively.

**Table:**

*D*—H···*A*	*D*—H	H···*A*	*D*···*A*	*D*—H···*A*
C6—H6···O1	0.95	2.50	2.886 (3)	105
C9—H9···O1	0.95	2.58	3.094 (4)	114
C15—H15A···N4	0.99	2.48	2.86 (2)	102
C15'—H15C···N2	0.99	2.47	2.84 (3)	102
C24—H24···O4	0.95	2.43	2.991 (3)	118
C6—H6···O4^i^	0.95	2.48	3.314 (3)	147
C20—H20···O3^ii^	0.95	2.46	3.386 (3)	164
C25—H25···Cg1^iii^	0.95	2.99	3.744 (3)	138
C15—H15A···Cg2^iv^	0.99	2.98	3.62 (2)	124

Symmetry codes: (i) *x*, *y*−1, *z*; (ii) *x*−1, *y*, *z*; (iii) −*x*, −*y*+1, −*z*; (iv) −*x*+1, −*y*+1, −*z*.
